# Dataset of permanent plots of trees with dbh >10cm in Mashpi rainforest biodiversity reserve, a remnant of the Chocó forest in Northern Ecuador

**DOI:** 10.1016/j.dib.2020.105845

**Published:** 2020-06-12

**Authors:** German Toasa, Carlos Morochz, Nora H. Oleas

**Affiliations:** a Cedral Ecolodge El Porvenir – Calacalí, Ecuador; b Reserva de Biodiversidad Mashpi, Quito, Ecuador; c Universidad Tecnológica Indoamérica, Centro de Investigación de la Biodiversidad y Cambio Climático (BioCamb) y Facultad de Ciencias de Medio Ambiente - Ingeniería en Biodiversidad y Recursos Genéticos, Machala y Sabanilla, CP. EC170103, Quito, Ecuador

**Keywords:** Biodiversity, Chocó forest, Forest survey, Tree

## Abstract

This data reports a list of all trees DBH > 10 cm in four 50 × 50 m (0.25 ha) permanent plots at Mashpi Rainforest Biodiversity Reserve in the Ecuadorian Chocó forest. Plots were established within an altitudinal gradient from 800 to 1200 m. We collected, labelled, measure and identify all trees found within the plots. All voucher specimens are available at the herbarium of Universidad Tecnológica Indoamérica (HUTI) in Quito, Ecuador. We found a total 133 stems representing 93 species and 36 families. Each plot had between 27 and 40 trees. Our list of species includes four threatened species under IUCN criteria. We also report the number of individuals of each species and its diameter at breast height (DBH) and height. This information is a baseline for further studies to contribute to the conservation of the Chocó, one of the 35 biodiversity hotspots is the Tumbes-Chocó-Magdalena. Even though this area is one of the most biodiverse in the planet, the botanical composition of the Chocó is still poorly known.

Specifications table

**Table:** 

Subject	Agricultural and Biological Sciences
Specific subject area	Forestry and Plant Science
Type of data	Table and Figure
How data were acquired	Data was obtained from April 10 to April 20, 2014. Our inventory consisted in four permanent 1/4 ha plots of 50 × 50 m each within the reserve. Plots were located within a distance of 900 to 1800 m with the aim of having a good representation of the altitudinal gradient in the area. We collected, labelled, and measure the diameter of all the trees with DBH ≥ 10 cm found within the plots. DHB was measure with a tape measure at 1.30 m taken from the base of the tree. Tree height was estimated by eye, because the canopy was too dense to calculate it with other methods. Each tree was marked with an aluminum tag, and the place where the DHB was measure was marked for future surveys. Herbarium specimens were deposited at the Herbario de la Universidad Tecnológica Indoaméria (HUTI) in Quito, Ecuador.
Data format	Raw
Parameters for data collection	Data collection considered trees of stem diameter ≥10 cm within permanent plots.
Description of data collection	Data was collected as part of the establishment of four permanent plots at Mashpi Rainforest Biodiversity Reserve, in Ecuador. Dataset comprises diameters at breast height (DBH) of all trees of stem diameter ≥ 10 cm found in the sampling area.
Data source location	Mashpi Rainforest Biodiversity Reserve, in Ecuador. Sampling plots names: PA: Puercos, PB: Limones, PC: San Vicente, PD: Malimpia. Plots geografic location: PA N 0.16077, W 78.87280 PB N 0.15870, W 78.88184 PC N 0.16712, W 78.86774 PD N 0.16849, W 78.87623
Data accessibility	With the article

## Value of the data

• Our sample represents a first survey of tree diversity at Mashpi Rainforest Biodiversity Reserve, a Chocó forest relict in Ecuador.
• Our results will serve to better understand the species diversity and conservation status of one of the remnant forests of the Ecuadorian Chocó.
• The generated data will be use as a baseline of further ecological studies such as carbon caption.

## 1 Data Description

Our dataset includes two tables. Table 1 compile a checklist of all the tree species and individuals found in each plot. A total of 133 stems of 92 species and 36 families were found. Each parcel of 0.25 ha has 27 to 40 trees with DBH > 10 cm. The most diverse family was Fabaceae with 10 species, followed by Lauraceae (7) and Rubiaceae (7). Most of the species were represented by one individual within the 0.25 ha plots. *Eschweilera caudiculata* (Lecythidaceae) was the only species with three individuals with DBH > 10 cm within one 0.25 ha plots. None of the species were found in all four plots. Only *Coussarea latifolia* (Rubiaceae), *Matisia castano* (Malvaceae) and *Naucleopsis capirensis* (Moraceae) were represented by four individuals combining all four plots. Among those species we found four threatened species under IUCN criteria. *Browneopsis macrofoliolata* (Fabaceae) is Critically Endangered (CR), under criteria A4c; B1ab(iii), *Inga silanchensis* (Fabaceae) is Vulnerable (VU) under criteria VU B1ab(iii), *Panopsis megistosperma* (Proteaceae) is Endangered (EN) under criteria EN B1ab(iv). We found one endemic species *Heisteria pacifica* (Olacaceae), which is Near Threatened NT.

**Table 1:** Checklist and number of individuals of tree species with DBH > 10 cm in four parcels of 0.25 ha in Mashpi Rainforest Biodiversity Reserve in the Ecuadorian Chocó forest. Sampling plots names: PA: Puercos, PB: Limones, PC: San Vicente, PD: Malimpia; IUCN: conservation status.

Taxon	PA	PB	PC	PD	Total	IUCN
Annonaceae		1		2	3	
Guatteria sp1.		1			1	
*Guatteria* sp2.				1	1	
*Rollinia pittieri* Saff.				1	1	
Aquifoliaceae	2				2	
*Ilex yurumanguinis* Cuatrec*.*	2				2	
Araliaceae			1		1	
*Dendropanax macrophyllus* Cuatrec*.*			1		1	
Arecaceae			1		1	
*Wettinia aequalis* (O.F. Cook & Doyle) R. Bernal			1		1	
Asteraceae			1		1	
*Critoniopsis occidentalis* (Cuatrec.) H. Rob.			1		1	
Boraginaceae		1	2	1	4	
*Cordia colombiana* Killip		1		1	2	
*Cordia cylindrostachya* (Ruiz & Pav.) Roem. & Schult.			1		1	
*Cordia lomatoloba* I.M. Johnst.			1		1	
Burseraceae	2			1	3	
*Dacryodes cupularis* Cuatrec*.*	2				2	
*Protium ecuadorense* Benoist				1	1	
Celastraceae	1			1	2	
*Salacia cordata* (Miers) Mennega	1			1	2	
Chrysobalanaceae	1		1		2	
*Licania durifolia* Cuatrec*.*	1		1		2	
Clusiaceae		1		1	2	
*Chrysochlamys dependens* Planch. & Triana				1	1	
*Garcinia macrophylla* Mart.		1			1	
Cyatheaceae			1		1	
*Cyathea caracasana* (Klotzsch) Domin			1		1	
Euphorbiaceae	1	2	2		5	
*Alchornea aff. grandis Benth.*			1		1	
*Croton pachypodus* G.L. Webster		1	1		2	
*Tetrorchidium euryphyllum* Standl.	1	1			2	
Fabaceae		8	2	3	13	
*Andira macrothyrsa* Ducke		1		1	2	
*Brownea coccinea* Jacq.		1			1	
*Browneopsis macrofoliolata* Klitg.		1			1	CR
*Dussia lehmannii* Harms			1	1	2	
*Inga aff. laurina* (Sw.) Willd.		1			1	
*Inga corruscans* Hum. & Bonpl. Ex Willd.		1	1		2	
*Inga punctata* Willd.		1			1	
*Inga silanchensis* T. D. Penn.		1			1	VU
*Inga spectabilis* (Vahl) Willd.		1			1	
*Zygia* sp.				1	1	
Icacinaceae		1	1	1	3	
*Calatola costaricensis* Standl.		1	1	1	3	
Lauraceae	3	3	1	4	11	
*Beilschmiedia costaricensis* (Mez & Pittier) C.K. Allen		1		1	2	
*Endlicheria formosa* (Meisn.) Mez				1	1	
*Licaria applanata* van der Werff		1			1	
*Nectandra* sp.		1			1	
*Ocotea insularis* (Meisn.) Mez	2			1	3	
*Pleurothyrium cinereum*			1	1	2	
*Rhodostemonodaphne kunthiana*	1				1	
Lecythidaceae	5	3	2		10	
*Eschweilera antioquensis*	2		1		3	
*Eschweilera caudiculata*		3			3	
*Eschweilera rimbachii*	1		1		2	
*Gustavia dodsonii*	1				1	
*Gustavia* sp.	1				1	
Malvaceae	1	4		1	6	
*Matisia castano*	1	2		1	4	
*Matisia giacomettoi*		2			2	
Melastomataceae		1	1	1	3	
*Loreya* sp.		1			1	
*Miconia brevitheca*				1	1	
*Miconia* sp. 1			1		1	
Meliaceae	1	2	2		5	
*Carapa nicaraguensis*		1			1	
*Guarea kunthiana*	1		1		2	
*Trichilia pallida*		1			1	
*Trichilia septentrionalis*			1		1	
Moraceae	1	4	4	2	11	
*Brosimum utile* subsp. *occidentale*		1			1	
*Ficus brevibracteata*	1		1		2	
*Ficus tonduzii*			2		2	
*Naucleopsis capirensis*		1	1	2	4	
*Naucleopsis naga*		2			2	
Myristicaceae	1	1			2	
*Otoba novogranatensis*	1				1	
*Virola calophylla*		1			1	
Myrtaceae	2	4		1	7	
*Calyptranthes brevispicata*	1				1	
*Calyptranthes* sp*.*		1			1	
*Eugenia crassimarginata*	1	2		1	4	
*Myrcia* sp*.*		1			1	
Olacaceae		1			1	
*Heisteria pacifica*		1			1	
*Pentaphylacaceae*	1				1	
*Freziera* sp*.*	1				1	
Phyllanthaceae	1	1		1	3	
*Hieronyma duquei*		1			1	
*Hieronyma fendleri*	1				1	
*Richeria grandis*				1	1	
Polygonaceae		1			1	
*Coccoloba obovata*		1			1	
Primulaceae	1	1	1		3	
*Ardisia croatii* subsp. *correae*			1		1	
*Geissanthus longistamineus*	1	1			2	
Proteaceae			1		1	
*Panopsis megistosperma*			1		1	EN
Rubiaceae	1	4	1	4	10	
*Borojoa aff patinoi*			1		1	
*Cordiera longicaudata*				1	1	
*Coussarea latifolia* Standl.		2		2	4	
*Duroia laevis*		1			1	
*Faramea langlassei*				1	1	
*Faramea parvibracteata*		1			1	
*Palicourea* sp.	1				1	
Sabiaceae		1			1	
*Meliosma occidentalis*		1			1	
Salicaceae			1		1	
*Banara guianensis*			1		1	
Sapindaceae	1			2	3	
*Allophylus floribundus*				1	1	
*Cupania livida*				1	1	
*Talisia macrophylla*	1				1	
Sapotaceae	3			2	5	
*Chrysophyllum colombianum*	1				1	
*Chrysophyllum* sp*.*				1	1	
*Pouteria collina*	2				2	
*Pouteria torta*				1	1	
Simaroubaceae				1	1	
*Simarouba amara*				1	1	
Tapisciaceae	1			1	2	
*Huertea glandulosa*	1			1	2	
Violaceae		1	1		2	
*Gloeospermum* aff*. grandifolium*		1	1		2	
Total of individuals	30	46	27	30	133	

In Table 2 we describe each individual tree collected per plot with a voucher number and DBH. The average overall DBH was 22.92 cm with a maximum of 93 cm of diameter.

**Table 2:** Plot inventory of tree species with DBH > 10 cm in four parcels of 0.25 ha in Mashpi Rainforest Biodiversity Reserve in the Ecuadorian Chocó forest.

Plot	Family	Species	Voucher	Plot number	DHB	H
PA	Aquifoliaceae	*Ilex yurumanguinis* Cuatrec.	Toasa12501	1	36	10
PA	Aquifoliaceae	*Ilex yurumanguinis* Cuatrec.	Toasa12510	24	17	16
PA	Burseraceae	*Dacryodes cupularis* Cuatrec.	Toasa12509	21	23	12
PA	Burseraceae	*Dacryodes cupularis* Cuatrec.	Toasa12513	33	11.7	12
PA	Celastraceae	*Salacia cordata* (Miers) Mennega	Toasa12522	72	77.5	30
PA	Chrysobalanaceae	*Licania durifolia* Cuatrec.	Toasa12502	2	34	16
PA	Euphorbiaceae	*Tetrorchidium euryphyllum* Standl.	Toasa12514	48	12.5	12
PA	Lauraceae	*Ocotea insularis* (Meisn.) Mez	Toasa12504	5	13	15
PA	Lauraceae	*Ocotea insularis* (Meisn.) Mez	Toasa12512	28	22	12
PA	Lauraceae	*Rhodostemono daphne* kunthiana (Nees) Rohwer	Toasa12511	25	33	30
PA	Lecythidaceae	*Eschweilera antioquensis* Dugand & Daniel	Toasa12505	7	17.5	8
PA	Lecythidaceae	*Eschweilera antioquensis* Dugand & Daniel	Toasa12524	76	11.6	7
PA	Lecythidaceae	*Eschweilera rimbachii* Standl.	Toasa12507	13	25	14
PA	Lecythidaceae	*Gustavia dodsonii* S.A. Mori	Toasa12521	67	18.5	13
PA	Lecythidaceae	*Gustavia* sp.	Toasa12523	73	39	18
PA	Malvaceae	*Matisia castano* H. Karst. & Triana	Toasa12520	65	15	8
PA	Meliaceae	*Guarea kunthiana* A. Juss.	Toasa12517	58	39	18
PA	Moraceae	*Ficus brevibracteata* W.C. Burger	Toasa12518	60	21.5	12
PA	Myristicaceae	*Otoba novogranatensis* Moldenke	Toasa12503	4	15	12
PA	Myrtaceae	*Calyptranthes brevispicata* McVaugh	Toasa12515	54	16.5	15
PA	Myrtaceae	*Eugenia crassimarginata* M.L. Kawas. & B. Holst	Toasa12526	80	18	4
PA	Pentaphylacaceae	*Freziera* sp.	Toasa12516	57	44.5	35
PA	Phyllanthaceae	*Hieronyma fendleri* Briq.	Toasa12508	15	12.5	20
PA	Primulaceae	*Geissanthus longistamineus* (A.C. Sm.) Pipoly	Toasa12506	11	12	7
PA	Rubiaceae	*Palicourea* sp	Toasa12528	87	11.5	4
PA	Sapindaceae	*Talisia macrophylla* (Mart.) Radlk.	Toasa12525	79	12	20
PA	Sapotaceae	*Chrysophyllum colombianum* (Aubrev.) T. D. Penn.	Toasa12527	82	17.5	4
PA	Sapotaceae	*Pouteria collina* (Little) T. D. Penn.	Toasa12626	8	41	22
PA	Sapotaceae	*Pouteria collina* (Little) T. D. Penn.	Toasa12627	71	54	30
PA	Tapisciaceae	*Huertea glandulosa* Ruiz & Pav.	Toasa12519	63	12.5	5
PB	Annonaceae	*Guatteria* sp1.	Toasa12539	114	31	22
PB	Boraginaceae	*Cordia colombiana* Killip	Toasa12559	158	13	12
PB	Clusiaceae	*Garcinia macrophylla* Mart.	Toasa12569	191	11.7	5
PB	Euphorbiaceae	*Croton pachypodus* G.L. Webster	Toasa12542	120	11.3	18
PB	Euphorbiaceae	*Tetrorchidium euryphyllum* Standl.	Toasa12548	131	10.7	10
PB	Fabaceae	*Andira macrothyrsa* Ducke	Toasa12541	119	13.7	8
PB	Fabaceae	*Brownea coccinea* Jacq.	Toasa12549	136	13.8	6
PB	Fabaceae	*Browneopsis macrofoliolata* Klitg.	Toasa12551	138	11.7	7
PB	Fabaceae	*Inga* aff *laurina* (Sw.) Willd.	Toasa12535	103	10.8	12
PB	Fabaceae	*Inga corruscans* Hum. & Bonpl. Ex Willd.	Toasa12564	181	19.8	12
PB	Fabaceae	*Inga punctata* Willd.	Toasa12628	89	37	27
PB	Fabaceae	*Inga silanchensis* T. D. Penn.	Toasa12545	124	10.8	7
PB	Fabaceae	*Inga spectabilis* (Vahl) Willd.	Toasa12631	155	37.7	25
PB	Icacinaceae	*Calatola costaricensis* Standl.	Toasa12557	156	11.5	4
PB	Lauraceae	*Beilschmiedia costaricensis* (Mez & Pittier) C.K. Allen	Toasa12558	157	29	12
PB	Lauraceae	*Licaria applanata* van der Werff	Toasa12546	126	36	25
PB	Lauraceae	*Nectandra* sp	Toasa12560	160	45	25
PB	Lecythidaceae	*Eschweilera caudiculata* R. Knuth	Toasa12533	97	16	8
PB	Lecythidaceae	*Eschweilera caudiculata* R. Knuth	Toasa12553	141	22.5	6
PB	Lecythidaceae	*Eschweilera caudiculata* R. Knuth	Toasa12570	192	13	8
PB	Malvaceae	*Matisia castano* H. Karst. & Triana	Toasa12543	121	18.5	8
PB	Malvaceae	*Matisia castano* H. Karst. & Triana	Toasa12565	183	25.5	15
PB	Malvaceae	*Matisia giacomettoi* Romero	Toasa12540	116	12	4
PB	Malvaceae	*Matisia giacomettoi* Romero	Toasa12563	180	11.5	6
PB	Melastomataceae	*Loreya* sp	Toasa12629	90	11.5	15
PB	Meliaceae	*Carapa nicaraguensis* C. DC.	Toasa12532	95	11.8	8
PB	Meliaceae	*Trichilia pallida* Sw.	Toasa12538	106	23.3	6
PB	Moraceae	*Brosimum utile* subsp. occidentale C.C. Berg	Toasa12547	130	42	30
PB	Moraceae	*Naucleopsis capirensis* C.C. Berg	Toasa12567	188	13	6
PB	Moraceae	*Naucleopsis naga* Pittier	Toasa12531	92	13.5	15
PB	Moraceae	*Naucleopsis naga* Pittier	Toasa12550	137	39	30
PB	Myristicaceae	*Virola calophylla* (Spruce) Warb.	Toasa12566	187	32	18
PB	Myrtaceae	*Calyptranthes* sp.	Toasa12555	149	14.8	10
PB	Myrtaceae	*Eugenia crassimarginata* M.L. Kawas. & B. Holst	Toasa12562	174	12.8	4
PB	Myrtaceae	*Eugenia crassimarginata* M.L. Kawas. & B. Holst	Toasa12552	140	15	8
PB	Myrtaceae	*Myrcia* sp	Toasa12571	194	11	7
PB	Olacaceae	*Heisteria pacifica* P. Jørg. & C. Ulloa	Toasa12534	101	14.5	5
PB	Phyllanthaceae	*Hieronyma duquei* Cuatrec.	Toasa12537	105	25.5	4
PB	Polygonaceae	*Coccoloba obovata* Kunth	Toasa12554	142	65.5	24
PB	Primulaceae	*Geissanthus longistamineus* (A.C. Sm.) Pipoly	Toasa12556	150	20	12
PB	Rubiaceae	*Coussarea latifolia* Standl.	Toasa12536	104	13.5	10
PB	Rubiaceae	*Coussarea latifolia* Standl.	Toasa12544	122	11.5	6
PB	Rubiaceae	*Duroia laevis* Devia, C.H. Perss. & C.M. Taylor	Toasa12561	163	21	12
PB	Rubiaceae	*Faramea parvibracteata* Steyerm.	Toasa12529	90	21	15
PB	Sabiaceae	*Meliosma occidentalis* Cuatrec.	Toasa12530	91	49	20
PB	Violaceae	*Gloeospermum* aff. *grandifolium* Hekking	Toasa12568	190	63	20
PC	Araliaceae	*Dendropanax macrophyllus* Cuatrec.	Toasa12596	319	28	15
PC	Arecaceae	*Wettinia aequalis* (O.F. Cook & Doyle) R. Bernal	Toasa12574	215	10	3
PC	Asteraceae	*Critoniopsis occidentalis* (Cuatrec.) H. Rob.	Toasa12588	266	92	12
PC	Boraginaceae	*Cordia cylindrostachya* (Ruiz & Pav.) Roem. & Schult.	Toasa12587	265	25	5
PC	Boraginaceae	*Cordia lomatoloba* I.M. Johnst.	Toasa12575	219	22.5	8
PC	Chrysobalanaceae	*Licania durifolia* Cuatrec.	Toasa12578	226	24.5	16
PC	Cyatheaceae	*Cyathea caracasana* (klotzsch) Domin	Toasa12633	233	11	3
PC	Euphorbiaceae	*Alchornea* aff. *grandis* Benth.	Toasa12577	223	32.3	15
PC	Euphorbiaceae	*Croton pachypodu*s G.L. Webster	Toasa12593	285	17	4
PC	Fabaceae	*Dussia lehmannii* Harms	Toasa12589	271	12	7
PC	Fabaceae	*Inga coruscans* Hum. & Bonpl. Ex Willd.	Toasa12595	316	22.5	15
PC	Icacinaceae	*Calatola costaricensis* Standl.	Toasa12576	220	17.5	6
PC	Lauraceae	*Pleurothyrium cinereum* van der Werff	Toasa12581	231	13	12
PC	Lecythidaceae	*Eschweilera antioquensis* Dugand & Daniel	Toasa12592	284	12.8	4
PC	Lecythidaceae	*Eschweilera rimbachii* Standl.	Toasa12573	207	33	18
PC	Melastomataceae	*Miconia* sp 1	Toasa12582	232	12.8	6
PC	Meliaceae	*Guarea kunthiana* A. Juss.	Toasa12580	230	11.5	8
PC	Meliaceae	*Trichilia septentrionalis* C. DC.	Toasa12583	242	10	5
PC	Moraceae	*Ficus brevibracteata* W.C. Burger	Toasa12586	262	25	6
PC	Moraceae	*Ficus tonduzii* Standl.	Toasa12584	247	16.2	14
PC	Moraceae	*Ficus tonduzii* Standl.	Toasa12591	278	19.2	10
PC	Moraceae	*Naucleopsis capirensis* C.C. Berg	Toasa12579	227	10.3	4
PC	Primulaceae	*Ardisia croatii* subsp. *correae* (Lundell) Ricketson & Pipoly	Toasa12585	261	36.8	12
PC	Proteaceae	*Panopsis megistosperma* Bonifaz & Cornejo	Toasa12594	301	15	8
PC	Rubiaceae	*Borojoa* aff *patinoi* Cuatrec.	Toasa12572	200	12.5	4
PC	Salicaceae	*Banara guianensis* Aubl.	Toasa12590	272	15.3	10
PC	Violaceae	*Gloeospermum* aff. *grandifolium*	Toasa12597	322	14	7
PD	Annonaceae	*Guatteria* sp2.	Toasa12612	367	93	20
PD	Annonaceae	*Rollinia pittieri* Saff.	Toasa12611	357	28.5	12
PD	Boraginaceae	*Cordia colombiana* Killip	Toasa12621	405	22	13
PD	Burseraceae	*Protium ecuadorense* Benoist	Toasa12618	386	33	14
PD	Celastraceae	*Salacia cordata* (Miers) Mennega	Toasa12606	336	23.3	8
PD	Clusiaceae	*Chrysochlamys dependens* Planch. & Triana	Toasa12624	415	20.1	5
PD	Fabaceae	*Andira macrothyrsa* Ducke	Toasa12610	352	21	8
PD	Fabaceae	*Dussia lehmannii* Harms	Toasa12604	334	36.2	15
PD	Fabaceae	*Zygia* sp	Toasa12636	362	13	3
PD	Icacinaceae	*Calatola costaricensis* Standl.	Toasa12609	346	14	8
PD	Lauraceae	*Beilschmiedia costaricensis* (Mez & Pittier) C.K. Allen	Toasa12602	331	25.5	8
PD	Lauraceae	*Endlicheria formosa* (Meisn.) Mez	Toasa12623	412	25	13
PD	Lauraceae	*Ocotea insularis* (Meisn.) Mez	Toasa12614	374	26	9
PD	Lauraceae	*Pleurothyrium cinereum* van der Werff	Toasa12601	330	24	20
PD	Malvaceae	*Matisia castano* H. Karst. & Triana	Toasa12600	327	11.1	3
PD	Melastomataceae	*Miconia brevitheca* Gleason	Toasa12598	325	10.3	4
PD	Moraceae	*Naucleopsis capirensis* C.C. Berg	Toasa12603	332	31.5	7
PD	Moraceae	*Naucleopsis capirensis* C.C. Berg	Toasa12617	382	14	8
PD	Myrtaceae	*Eugenia crassimarginata* M.L. Kawas. & B. Holst	Toasa12615	375	11.5	6
PD	Phyllanthaceae	*Richeria grandis* Vahl	Toasa12619	388	10.5	4
PD	Rubiaceae	*Cordiera longicaudata* C.H. Perss. & Delprete	Toasa12622	407	11	5
PD	Rubiaceae	*Coussarea latifolia* Standl.	Toasa12599	326	16.6	5
PD	Rubiaceae	*Coussarea latifolia* Standl.	Toasa12605	335	33.3	10
PD	Rubiaceae	*Faramea langlassei* Standl.	Toasa12607	337	14	5
PD	Sapindaceae	*Allophylus floribundus* (Poepp.) Radlk.	Toasa12620	389	11	4
PD	Sapindaceae	*Cupania livida* (Raldk.) Croat	Toasa12625	428	57.5	20
PD	Sapotaceae	*Chrysophyllum* sp	Toasa12635	356	15	8
PD	Sapotaceae	*Pouteria torta* (Mart.) Radlk.	Toasa12613	372	13	7
PD	Simaroubaceae	*Simarouba amara* Aubl.	Toasa12616	376	45	30
PD	Tapisciaceae	*Huertea glandulosa* Ruiz & Pav.	Toasa12608	339	15	6

## 2 Experimental design, materials, and methods

Our survey was conducted in the Bosque Protector Mashpi, a Natural Forest Reserve declared in September 2003. The Reserve has 1178 ha [1] and it is in the Mashpi watershed at the Pichincha province in Ecuador [2]. The area is characterized by deep slopes of 45° to 90°, which might have helped to conserve the forest from logging activities [2].

**Fig. 1:**
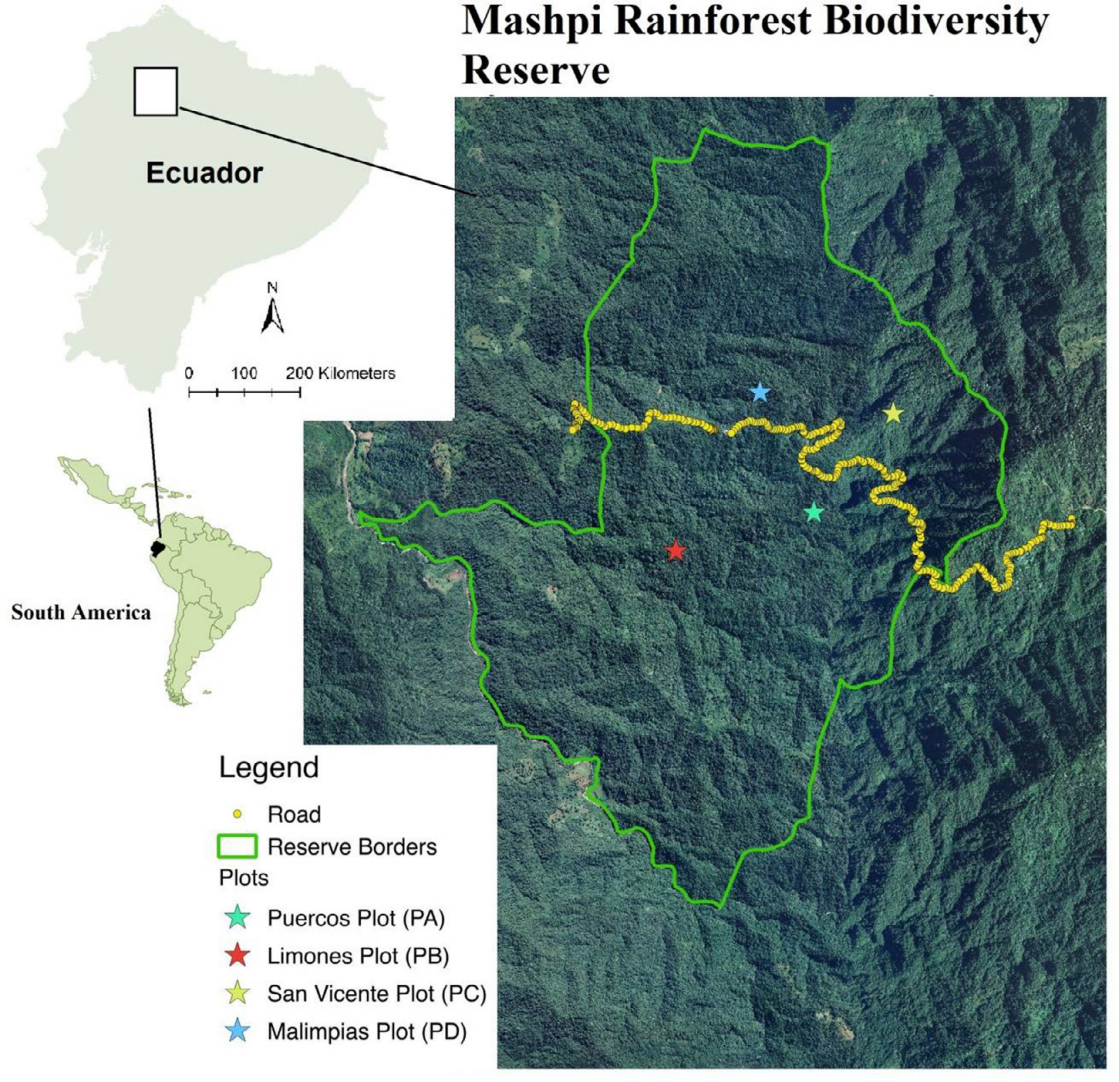
Map of the geographic distribution of the permanent plots of trees with DBH > 10  in the Mashpi Rainforest Biodiversity Reserve, a remnant of the Chocó in Northern Ecuador.

**Fig. 2:**
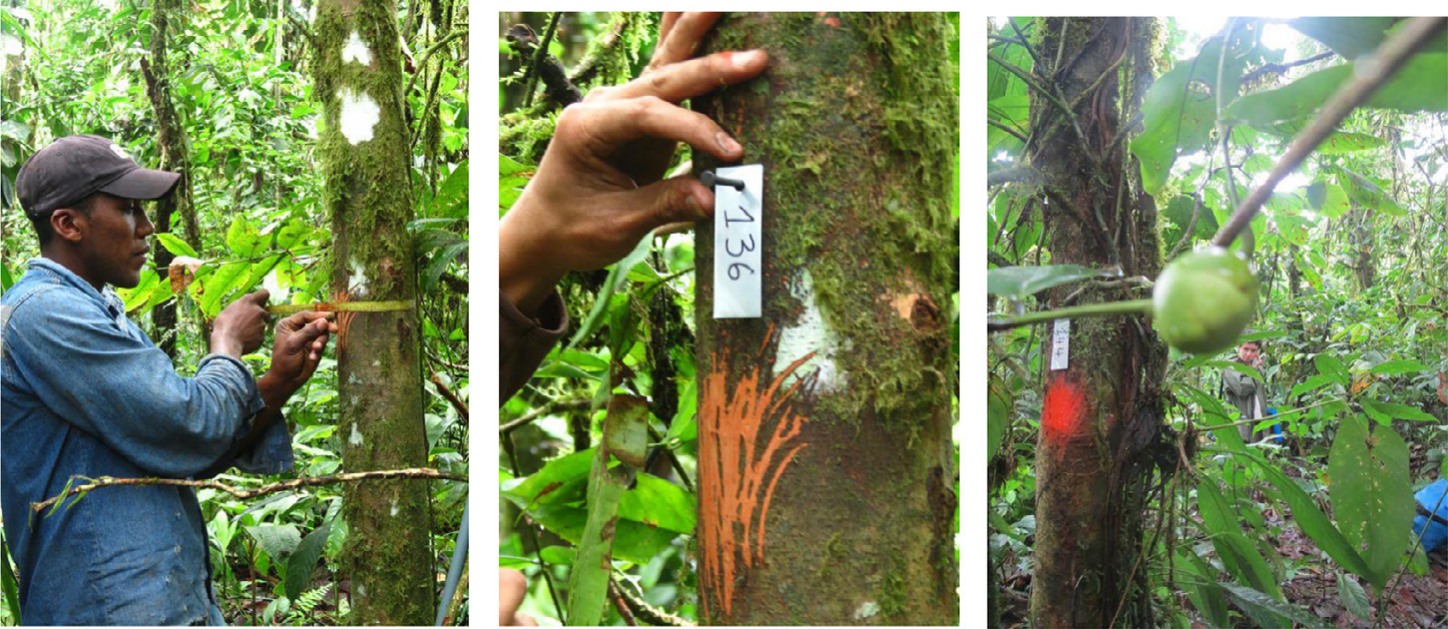
DHB sampling and label mark for each tree at the survey plot at Masphi Rainforest Biodiversity Reserve.

Our inventory consisted in four permanent 1/4 ha plots of 50 × 50 m each within the reserve. Plots were limited with rope tied to 1/4 inch PVC tubes painted in orange. The specific locations are listed in Table 3 (Fig. 1). Plots were located within a distance of 900 to 1800 m and they were set to have a good representation of the altitudinal gradient in the area. We collected, labelled, and measured the diameter of all the trees with DBH ≥ 10 cm found within the plots. DBH was measure at 1.30 m from the base of the tree, following methods by Campbell [3]. Tree height was estimated by eye (by Toasa), because the canopy was to dense to calculate it with other methods. Each tree was marked with an aluminum tag, and the place where the DHB was measure was marked for future surveys (Fig. 2). Specimens were identified by Germán Toasa comparing vouchers at QCNE Herbarium in Quito. The herbarium specimens were deposited at the Herbario de la Universidad Tecnológica Indoaméria (HUTI) in Quito, Ecuador. Scientific names followed APG IV [4] and were checked at http://www.tropicos.org/ [5].

**Table 3:** Summary of the plots surveyed in Mashpi Rainforest Biodiversity Reserve, Chocó, Northern Ecuador.

Plot name	Coordinates	Altitude (m)	Slope(%)	No. Individuals (stems)	Species number	Average DHB (cm)	Max DHB (cm)
PA	N 0.16077, W 78.87280	952	50	30	25	24.46	77.5
PB	N 0.15870, W 78.88184	846	40	46	40	22.12	65.5
PC	N 0.16712, W 78.86774	1186	15	27	26	21.17	92
PD	N 0.16849, W78.87623	1018	40–50	30	28	24.16	93
Mean				33.25	23	22.98	82
Total				133	92

This is the first survey of trees implemented in the Reserve. The plots were stablished during one fieldtrip from April 10 to April 20, 2014. Our plots represent a first approximation to estimate tree diversity at Mashpi Rainforest Biodiversity Reserve, a Chocó relict in Ecuador. Our results will serve as a base line to better understand the species diversity and conservation status of one of the relict forests of the Ecuadorian Chocó. The Reserve is managed by Mashpi Lodge https://www.mashpilodge.com/. Some of the studies associated with the plots are: Potential food abundance for reintroduction of *Ateles fusciceps* “spider monkey”, and CO2 absorption data [6].

## Declaration of Competing Interest

None.
